# Crystallization of ApoA1 and ApoE4 nanolipoprotein particles and initial XFEL-based structural studies

**DOI:** 10.3390/cryst10100886

**Published:** 2020-10-01

**Authors:** M.L. Shelby, D. Gilbile, T.D. Grant, W.J. Bauer, B. Segelke, W. He, A.C. Evans, N. Crespo, P. Fischer, T. Pakendorf, V. Hennicke, M.S. Hunter, A. Batyuk, M. Barthelmess, A. Meents, T.L. Kuhl, M. Frank, M.A. Coleman

**Affiliations:** 1 Biosciences and Biotechnology Division, Lawrence Livermore National Laboratory, Livermore, CA, USA.; 2 Department of Chemical Engineering, University of California at Davis, Davis, CA, USA.; 3 Department of Structural Biology, Jacobs School of Medicine and Biomedical Sciences, SUNY University at Buffalo, Buffalo, NY, USA; 4 Hauptman-Woodward Medical Research Institute, Buffalo, NY, USA.; 5 Center for Free-Electron Laser Science, Hamburg, Germany.; 6 Linac Coherent Light Source, SLAC National Accelerator Laboratory, Menlo Park, California, USA.

**Keywords:** lipoprotein, nanodisc, serial femtosecond crystallography, XFELs, fixed target delivery

## Abstract

Nanolipoprotein particles (NLPs), also called “nanodiscs”, are discoidal particles with a patch of lipid bilayer corralled by apolipoproteins. NLPs have long been of interest due to both their utility as membrane-model systems into which membrane proteins can be inserted and solubilized and their physiological role in lipid and cholesterol transport via HDL and LDL maturation, which are important for human health. Serial femtosecond crystallography (SFX) at X-ray free electron lasers (XFELs) is a powerful approach for structural biology of membrane proteins, which are traditionally difficult to crystallize as large single crystals capable of producing high-quality diffraction suitable for structure determination. To facilitate understanding of the specific role of two apolipoprotein/lipid complexes, ApoA1 and ApoE4, in lipid binding and HDL/LDL particle maturation dynamics and develop new SFX methods involving NLP membrane protein encapsulation, we have prepared and crystallized homogeneous populations of ApoA1 and ApoE4 NLPs. Crystallization of empty NLPs yields semi-ordered objects that appear crystalline and give highly anisotropic and diffuse X-ray diffraction, similar in characteristics to fiber diffraction. Several unit cell parameters were approximately determined for both NLPs from these measurements. Thus, low-background, sample conservative methods of delivery are critical. Here we implemented a fixed target sample delivery scheme utilizing the Roadrunner fast-scanning system and ultra-thin polymer/graphene support films, providing a low-volume, low-background approach to membrane protein SFX. This study represents initial steps in obtaining structural information for ApoA1 and ApoE4 NLPs and developing this system as a supporting scaffold for future structural studies of membrane proteins crystalized in a native lipid environment.

## 1. Introduction

The Nanolipoprotein particles (NLPs), or “nanodiscs”, are nanoscale complexes with apolipoproteins forming a corralled, discoidal bilayer.^1^ There are many biotechnological applications of NLPs, including the use of NLPs for structural characterization of transmembrane proteins, which have been historically intractable to X-ray crystallography and other structural techniques due to their low solubility and tendency to aggregate. Using membrane mimetics that solubilize and facilitate functional studies of membrane proteins via X-ray scattering, CryoEM, or X-ray crystallography has been extensively demonstrated by us and others.^2–15^ Discoidal lipoproteins, which are closely related to NLPs, represent a critical physiological transition state from lipid-free apolipoprotein to spherical HDL and LDL particles during HDL/LDL maturation, and thus play a role in the risk of cardiovascular disease, protection against atherosclerosis, and amyloid related diseases due to their key role in reverse cholesterol transport (RCT).^16, 17^

Interaction between nascent HDL/LDL discs and lecithin cholesterol acyltransferase (LCAT) leads to conversion of cholesterol to cholesteryl ester and apolipoprotein mediated formation of the spherical particles.^18–23^ Current structural models of the disc suggest an anti-parallel apolipoprotein chain orientation surrounding the lipid bilayer patch that can adjust to accommodate a number of discrete particle diameters based on the protein to lipid ratio. Computational modeling has been performed in the presence of phospholipids^24^ and several NMR solution structures exist.^25, 26,27^ The apolipoprotein-mediated lipid binding properties of HDL/LDL and the importance of specific sub-populations of nascent HDL implies control by the apolipoprotein over the maturation process.^28, 29^ Therefore, a mechanistic understanding of HDL/LDL maturation and its role in disease states requires an understanding of how proteins involved in HDL maturation, such as LCAT, interact with the disc and of how apolipoprotein-lipid and apolipoprotein-cholesterol/ cholesteryl ester interaction controls final particle size. We have optimized protocols to generate homogenous preparations of NLPs^1, 24, 30^ for structural studies confirmed by small angle x-ray scattering (SAXS) and small angle neutron scattering (SANS).^10, 31, 32^

**Figure 1.**
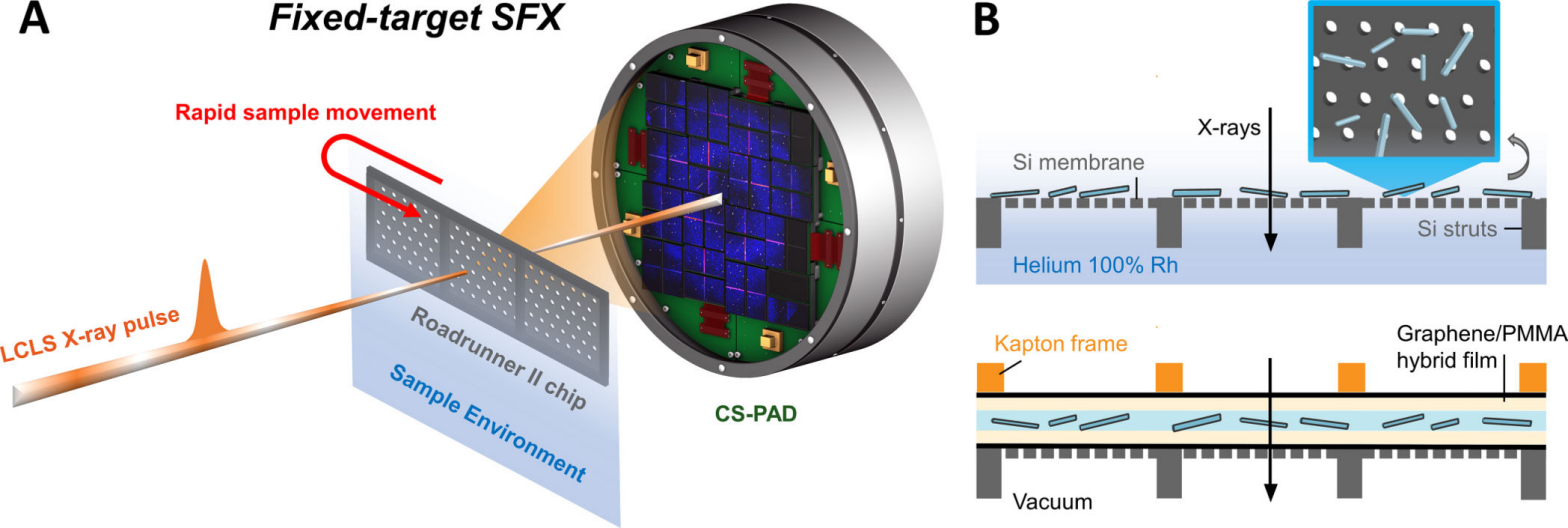
Scheme illustrating A) SFX data collection and the fixed target sample delivery approach using B) crystal deposition onto a second-generation Roadrunner II micropatterned Si chips. Cross sections (not to scale) of chips used at MFX at near 100% relative humidity (above) and the Graphene/PMMA enclosed chips used at CXI in vacuum (below) are shown.

Several X-ray crystallographic studies of lipid-bound apolipoproteins at low resolution have been published that contain diffuse features or weak diffraction and report a high degree of susceptibility to radiation damage even at cryogenic temperature.^33, 34^ Serial femtosecond crystallography (SFX) combined with low-background fixed-target sample delivery (Figure 1) is well suited to mitigate fast crystal decay due to radiation damage. The ultrashort, high brightness pulses from XFELs, such as the Linac Coherent Light Source (LCLS), can capture damage-free,^35^ single-pulse diffraction images from biological micro- and nano-objects at room temperature, including protein microcrystals^36, 37^ and weakly diffracting objects like virus particles,^38, 39^ 2-dimensional protein crystals,^40–42^ or protein fibrils.^43^ Using the SFX approach, high-resolution macromolecular structures can be derived from thousands of individual diffraction patterns obtained sequentially from continuously replenished microcrystals.^44–46^ A fixed target sample delivery approach can drastically reduce the amount of sample required for experiments limited by protein or crystallization yield by minimizing crystal loss to, for example, jet flow, while enabling the measurement of samples with heterogeneous mixtures of crystal size. The “Roadrunner” fast scanning fixed target system developed by the Centre for Free-Electron Laser Science (CFEL) utilizes fast stages synchronized with the X-ray pulse arrival time such that X-rays shots align with the pores of a micropatterned silicon chip^47^ at the 120 Hz repetition rate of LCLS.^48^ To enable fixed target SFX in-vacuum, we have recently demonstrated the use of large-area few-layer graphene (FLG) in conjunction with polymer thin-films, as enclosing layers to maintain sample hydration for room temperature studies, while imparting mechanical robustness and only minimally adding to the X-ray scatter background.^49^

**Figure 2.**
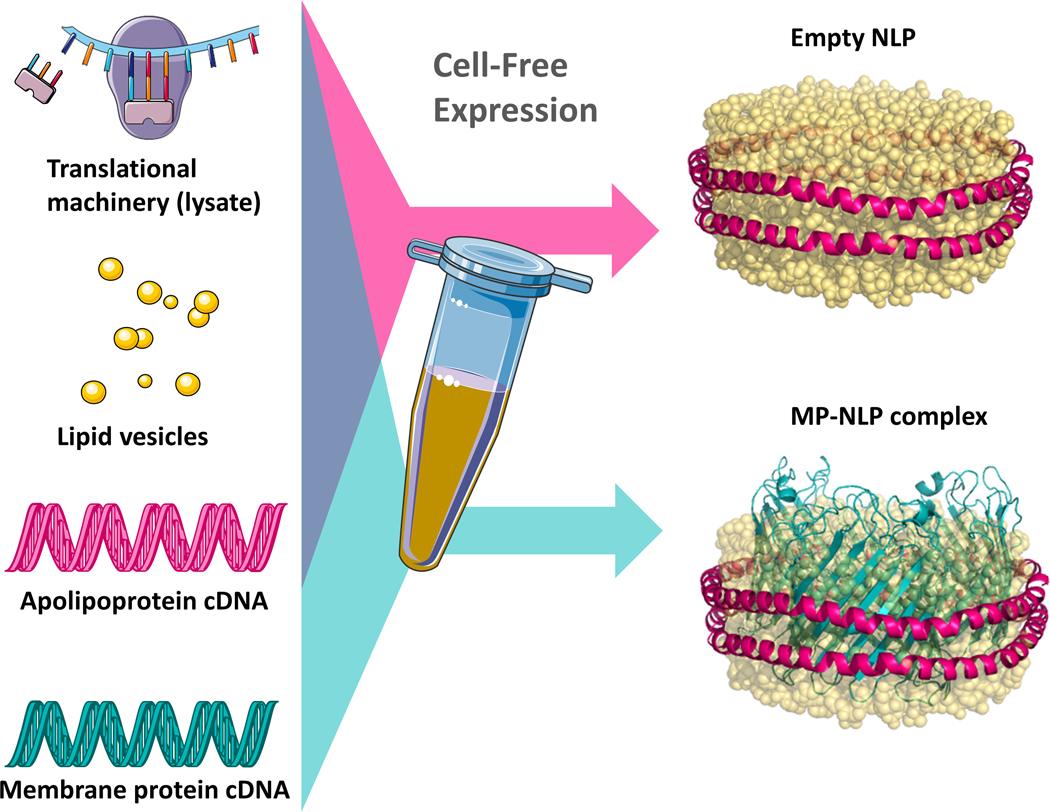
Cell-free expression and self-assembly of empty NLPs (pink arrow) and NLPs with embedded transmembrane proteins (teal arrow).

To understand the structure and function of lipoprotein particles and NLPs, we have optimized crystallization protocols for multiple types of lipid-bound apolipoproteins (ApoA1 and ApoE4) to be subject to SFX at LCLS. Our lipid-bound apolipoproteins are produced via cell-free expression (Figure 2). Cell free expression is an optimal route to assembly of NLPs and scaffold supported membrane proteins of interest due to its open architecture and elimination of the need for detergent solubilization or extensive purification.^50–53^ This provides a path forward for understanding the formation of mature spherical forms of HDL/LDL particles and constitutes a critical step in validating membrane protein-NLP complexes as a platform for structural studies.

## 2. Materials and Methods

### 2.1. Cell-free expression, preparation, and characterization of ApoA1 and ApoE4 nanolipoprotein particles

Preparative 1 ml reactions were carried out using the Rabbit Biotech RTS 500 ProteoMaster *E. coli* HY Kit for protein expression and purification as previously described.^32^ In brief, lyophilized reaction components (*E. coli* lysate, reaction mixture, amino acid mixture, and methionine) were dissolved in reconstitution buffer and combined as specified by the manufacturer. A total of 1 μg of plasmid DNA, either the truncated forms of apolipoprotein A-I (Δ1–49), or apolipoprotein E-4 (22k), was added to the lysate mixture along with 1,2-dimyristoyl-sn-glycero-3-phosphocholine (DMPC) vesicles. Small unilamellar vesicles (SUV) below 100 nm in size of DMPC were prepared by probe-sonicating a 20 mg/ml aqueous solution of DMPC until optical clarity was achieved. The SUVs were added to the cell-free reaction at a concentration of 2 mg/ml. The reactions were incubated at 37 °C overnight for approximately 18 hrs. Immobilized nickel affinity chromatography was used to isolate the His-tagged apolipoproteins according to the manufacturer’s protocol (Roche Molecular Diagnostics). Elution fractions were assayed with SDS-PAGE and peak elution fractions containing the protein of interest were pooled and buffer-exchanged overnight into 20 mM ammonium bicarbonate pH 7.0 using a 10 kDa molecular weight sieve. The pooled protein was then concentrated with 10 kDa molecular weight cutoff spin concentrators to 5 mg/ml in preparation for crystallization experiments and stored in 20 mM ammonium bicarbonate pH 7.0. Dynamic Light Scattering (DLS) measurements were performed on diluted samples of the ApoA1 and ApoE4 discs with a Zetasizer Nano Particle Size Analyzer (Malvern Panalytical).

### 2.2. High-throughput crystallization screening

ApoA1 and ApoE4 NLP solutions at 5 mg/ml protein concentration in 20 mM ammonium bicarbonate were submitted to the high throughput crystallization screen at the High-Throughput Screening Laboratory at the Hauptman-Woodward Medical Research Institute in Buffalo, New York. This facility makes use of automated liquid handling and imaging systems coordinated through remote-accessible database to quickly set up and record the outcomes of 1,536 unique microbatch-under-oil crystallization screening experiments per submission. 400 nl crystallization drops were dispensed under paraffin oil and monitored over six weeks via optical imaging. All wells were imaged with Ultraviolet Two-Photon Excited Fluorescence (UV-TPEF) to identify biological crystalline objects via tryptophan fluorescence and second-order nonlinear optical imaging of chiral crystals (SONICC) at 28 days.

### 2.3. Preparation of NLP crystals for SFX experiments

Selected crystal hits identified in the high throughput screen (Table S1) were independently reproduced in microbatch-under-oil experiments (Table 1). NLP solutions concentrated to 5 mg/ml in protein were mixed in 1 μl quantities with several volume ratios of each cocktail (typically 2:1, 1:1, and 1:2) in Terasaki-style microbatch trays and covered with a thin layer of paraffin oil. Wells were incubated at 21°C and monitored for crystal growth with optical microscopy over a four-week period. Several crystallization conditions showing apparent crystal growth were selected and optimized to maximize crystal number, density, and uniformity for SFX experiments. Crystals for SFX experiments were grown in a large number of replicate wells in microbatch trays under paraffin oil using crystallization cocktails summarized in Table 1, harvested from individual wells, and pooled to yield at least a 50 μl volume of crystal slurry for deposition onto the micropatterned chips. For conditions with low crystal density, the slurry was concentrated two-fold with gentle spin centrifugation.

### 2.4. Fixed target preparation and sample deposition

SFX experiments took place over two 6-hour Protein Crystal Screening beamtimes at the MFX and CXI endstations at LCLS using micropatterned silicon-based fixed targets for sample delivery (Figure 1). Micro-patterned single crystalline silicon chips were prepared at CFEL or commercially manufactured by Finnlitho (Joensuu, Finland) following the design principles of second generation Roadrunner chips described previously.^54^ Each chip (32.7 mm x 12 mm) used at MFX was comprised of a 200 μm thick frame with three 10 mm x 10 mm square areas of Si “windows” thinned to 10 μm and supported by 100 μm wide struts in between the windows (Figure S1). These windows were patterned further with a hexagonal dense pattern of 15 μm pores spaced 100 μm apart, yielding >40,000 pores per chip that could hold crystal samples. Chips used at CXI had a similar design but had a slightly different window configuration and pore spacing (18 × 5 array of 1.5 mm x 1.5 mm windows with 50 μm pore spacing).

Experiments were conducted in humidified atmosphere, Roadrunner III system at MFX, or in vacuum using the vacuum-compatible Roadrunner IV system in the 0.1 μm *in vacuo* sample environment of CXI, necessitating two approaches to chip preparation and sample deposition. For humidified environment experiments at MFX, Finnlitho chips were loaded with 50 μl of freshly crystallized NLP microcrystal suspension by pipetting and spreading the crystal slurry onto the flat side of the chip and wicking away excess mother liquor from the opposite side to aid in drawing microcrystals into the chip pores. The loaded chips were then immediately transferred to the Roadrunner sample chamber flushed with humidified helium (near 100 % humidity).

Preparation of the graphene-polymer film “sandwich” used to preserve sample hydration in vacuum at CXI was performed as described in Shelby and Gilbile et. al., 2020.^49^ A silicon chip was covered on the flat side by a film comprised of few-layer graphene (FLG) and 40 nm thick polymethyl methacrylate (PMMA) and approximately 20 μl of a two-fold concentrated S816–4 ApoA1 microcrystal slurry was carefully pipetted onto the film. A Kapton frame with a secondary PMMA-FLG film was then carefully aligned with the chip and placed on top of the microcrystal solution to aid in solution spread over the chip area by capillary action. The edges of the sandwiched sample were sealed by application of a thin layer of vacuum grease to prevent dehydration. Chips prepared with ApoA1 NLP microcrystals were tested for hydration retention in a vacuum chamber at room temperature prior to the SFX experiment. Figure S2 shows intact crystals after 30 minutes in the vacuum chamber.

### 2.5. SFX experiments

SFX experiments were conducted at an X-ray energy of 7.5 keV and 9.5 keV with a beam size at the sample of 120 nm x 170 nm full width half maximum (FWHM) and 3 μm x 3 μm FWHM for experiments at CXI^55^ and MFX (humidified atmosphere), respectively. During each sample scan, chips were translated through the beam at the full 120 Hz repetition rate of LCLS such that the X-ray pulse arrival was synchronized spatially with the patterned micropores and diffraction images were recorded shot-by-shot by a Cornell-SLAC Pixel Array Detector (CSPAD).^56^ The design and operation of the Roadrunner fast scanning system is described in detail elsewhere.^47–49, 57 58^ NLP samples were measured with between 3 % and 10 % beam transmission (at CXI) and between 20 and 30% beam transmission (at MFX) rather than the full X-ray flux of 4.5 mJ/pulse due to concerns regarding damage to the chip resulting from the lower-intensity “wings” of the X-ray beam around the central focus spot (~1 % of the total intensity). All chips were assessed for damage immediately following measurement with optical microscopy. The nominal pulse duration was 40 fs for both experiments. During MFX experiments, an aluminum attenuator plate covered the low-resolution area of the detector to a resolution of ~6 Å.

X-ray images were analyzed in near real-time for an estimation of hit rate using OnDA^59^. Cheetah^60^ was used to find crystal hits. ADXV^61^ was used to visualize hits and to measure inter-reflection spacing by integrating signal intensity over ± 3pixels along lattice lines.

## 3. Results and Discussion

### 3.1. Cell-free expression

Cell-free expression and assembly of ApoA1 and ApoE4 was selected as a preparative strategy for crystallization. This is based on both its established capabilities to produce high yields of homogeneously sized NLPs and to facilitate future structural studies of membrane proteins through co-translational techniques that enable facile insertion of membrane proteins into the NLP bilayer.^62^ Previous studies have extensively characterized the particle size and homogeneity resulting from our cell free assembly. Samples were characterized with: size exclusion chromatography (SEC), DLS, negative stain TEM, and SAXS/SANS.^27, 32, 53, 62–66^ The yields of protein ranged from 3–4 mg of apolipoprotein, which were typical for a 1 ml cell-free reaction. Based on SDS-PAGE analysis the purified protein was greater than 98% homogeneous. To assess NLP formation and size distribution, we ran DLS, which confirmed that the pooled Ni-NTA affinity purified NLPs were homogenously distributed in size, centered at 11.5 nm and 32.0 nm for ApoA1 and ApoE4 NLPs respectively (Figure S3). NLP preparations were deemed sufficient for crystallization screens based on both the high yield and purity.

### 3.2. Crystallization Optimization

**Figure 3.**
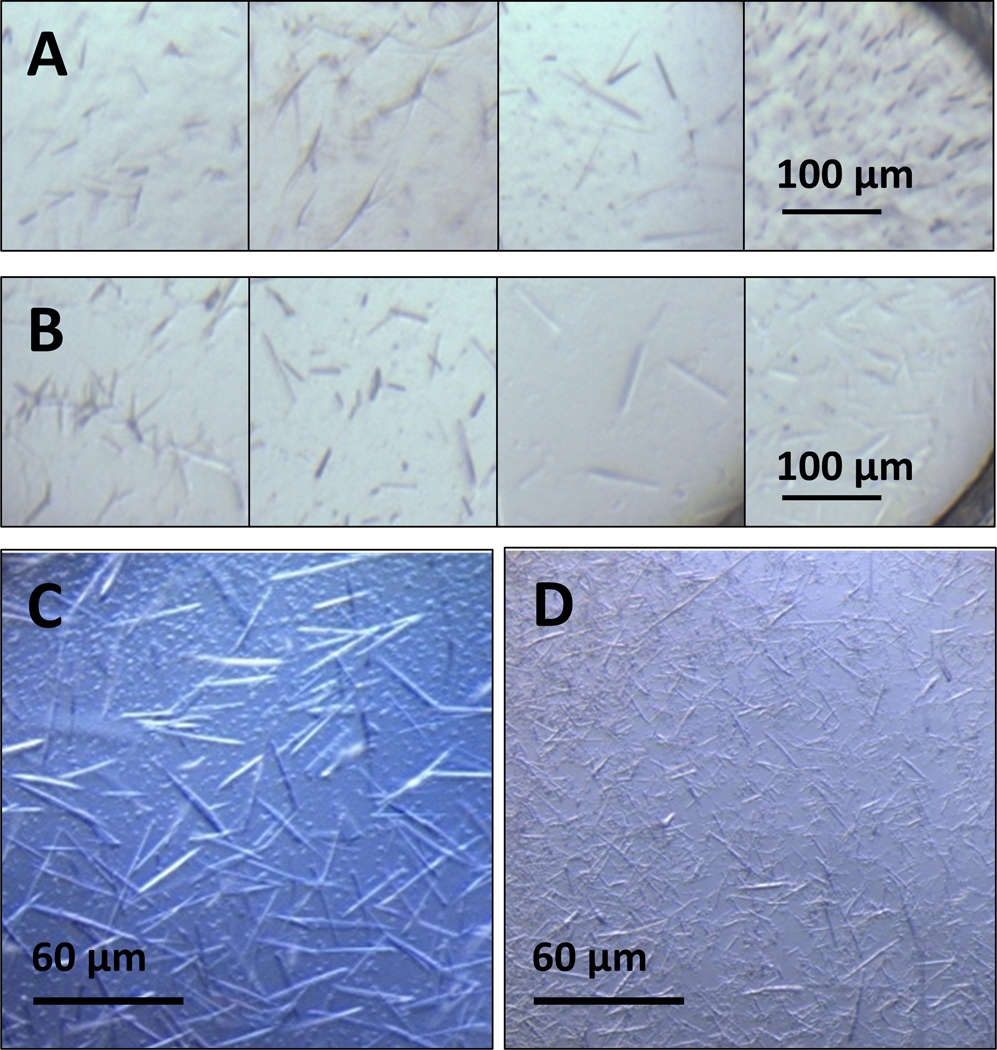
Hits generated by the HWI High throughput screen for A) ApoA1 and B) ApoE4 NLPs. Wells were imaged between 2 and 4 weeks after the experiment was initiated using a custom-built optical imaging system at 21°C. Rod/rod cluster or needle/needle cluster morphologies dominate hit conditions containing varying buffer and pH conditions, salt additives, and precipitants for both ApoA1 and ApoE4 NLPs. Hits reproduced and optimized for SFX at LLNL for C) ApoA1 and D) ApoE4. Conditions pictured are C) S816–4 and D) M1328.

Initial high throughput crystallization screens subjected both NLP types to a wide range of crystallization conditions to obtain crystal hits for further optimization. Screens designed for both soluble proteins^67^ and membrane proteins^68^ were applied for a total of 2976 unique cocktails. The screens included incomplete factorial experiments consisting of PEGs and other polymer precipitants of varying molecular weight, salts, and a variety of pHs and buffer conditions, as well as commercially available screens from Hampton Research (including PEGRx HT, PEG Ion HT, Crystal Screen HT, Index, Salt Rx HT, Silver Bullet with PEG3350 pH 6.8 precipitants, Grid Screen Ammonium Sulfate, Slice pH, Ionic Liquids, and Polymer Screen) and Molecular Dimensions (MemGold screen) used as received or lightly modified.^67, 68^ Hits were identified from manual scoring of optical imaging of individual wells at day one and subsequently at weekly intervals. Potential hits identified with optical imaging were compared to UV-TPFF imaging at 28 days to confirm that crystalline objects were protein-containing rather than inorganic material. This scoring process identified a total of 165 and 237 potential hits for the ApoA1 and ApoE4 NLP samples respectively and are summarized in Table S1 and Figure S4. The dominant morphologies among hits for both proteins were either individual rods or needles or clusters of rods or needle-like objects (Figure 3) and the majority of hits were detected after relatively long incubation periods, typically at the three-week timepoint. Some trends were identified in the hits obtained, particularly the prevalence of mono or divalent inorganic salts, mid-range or very low molecular weight PEGs, and pH conditions at or above the isoelectric point of the apolipoprotein (5.56 and 5.65 for ApoA1 and ApoE4 respectively^69^) (Figure S4).

Of these, 10 conditions for each apolipoprotein were successfully reproduced and further optimized for SFX experiments from a subset of hits selected to explore a large chemical space and for apparent crystal quality. In general, a condition that produces a high density of uniform crystals on the scale of tens of micron, as opposed to a few crystals on the 100s of micron scale, is ideal for SFX and conditions were optimized as judged by reproducibility, crystal density, and uniformity. The most successful conditions, summarized in Table 1, chiefly consist of high concentrations of mid molecular weight PEGs (~20–40% w/v PEG 1000 or 4000) and salts with monovalent anions (Li^+^, Na^+^, NH_4_^+^). For ApoA1, conditions were primarily at pH 10 with CAPS while conditions for ApoE4 were optimized at both relatively low pH (sodium citrate, pH 5.5–5.6) and well above the ApoE4 pI (Tris pH 8.5). Crystal size distributions and number densities were measured by counting optical images of known volumes of crystal slurry for the conditions with the four highest apparent number densities for each apolipoprotein (Table 1).

### 3.3. SFX Experiments

**Figure 4.**
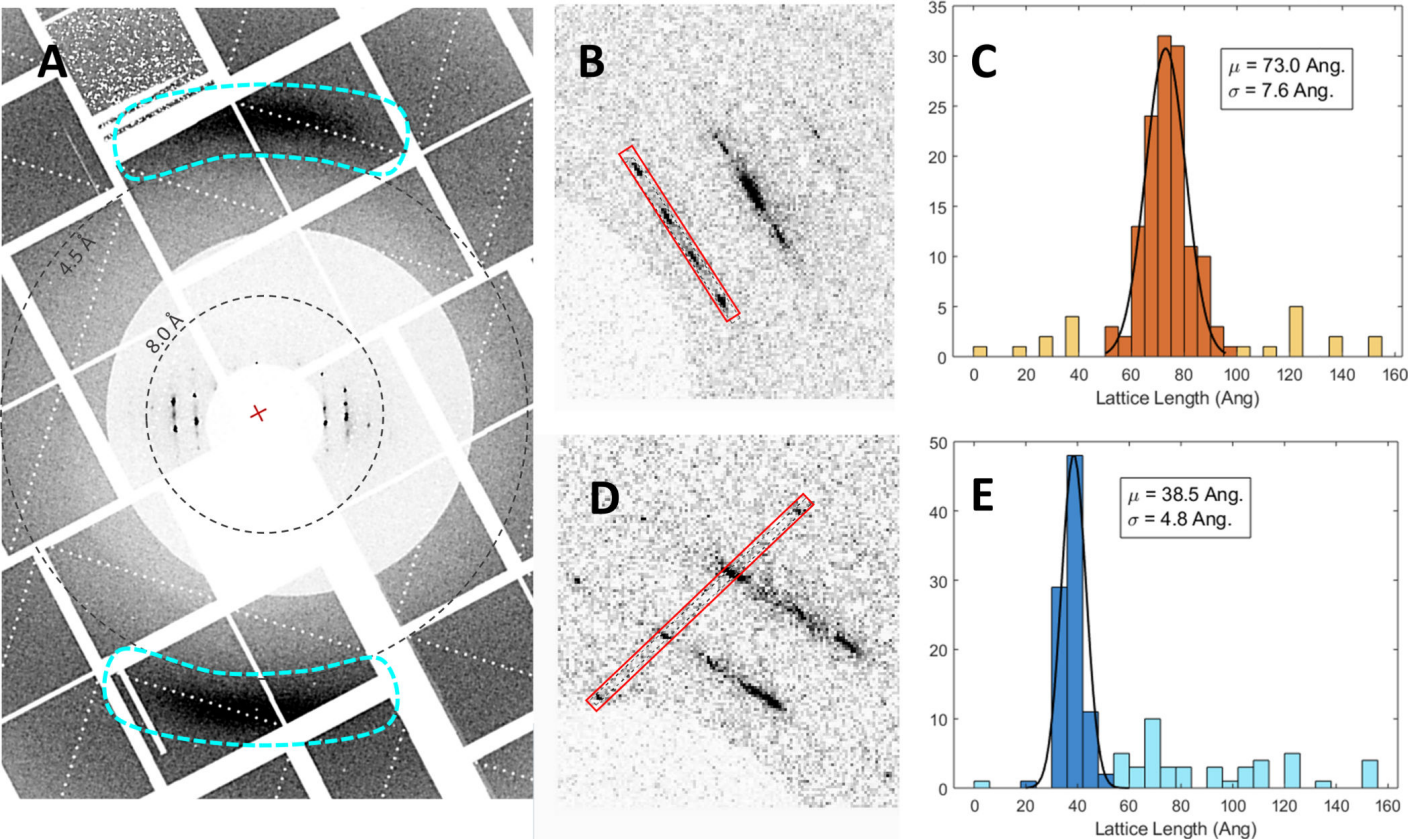
An example of hits collected at MFX for S816–4 ApoA1 at MFX showing A) anisotropic diffraction and diffuse features at 4.2 Å, and averaging of signal intensities along B) layer lines and D) an orthogonal crystallographic axis. The equivalent histograms of the reflection spacing measured from B) and D) are shown in C) and E) respectively.

**Figure 5.**
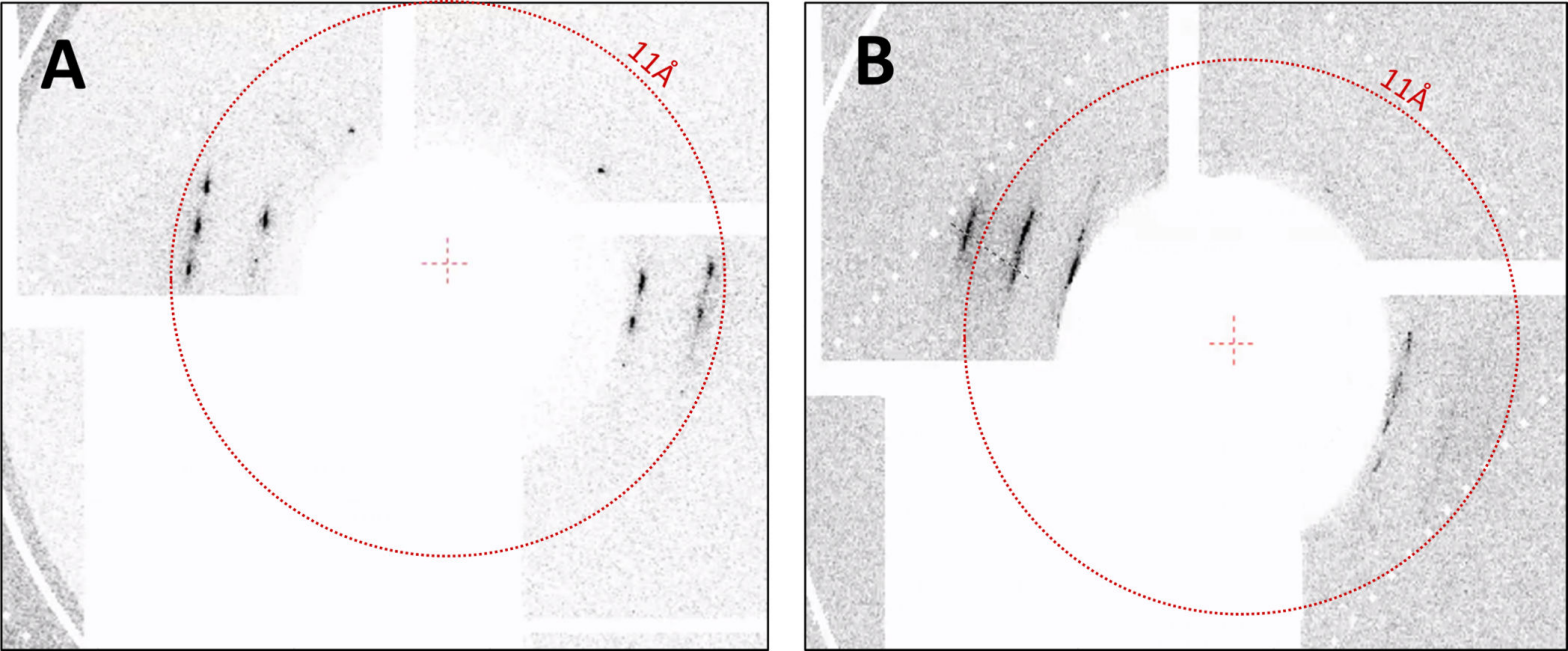
An example of hits collected at MFX for A) S816–4 ApoA1 and B) S1022 ApoE4 showing differences in diffusivity of peaks within layer lines at low resolution.

Crystals from three conditions were measured in the humidified helium environment at MFX, S816–4 (ApoA1), S1022 (ApoE4) and S1328 (ApoE4) (Table 1, boxed conditions). CSPAD images from ~42,900 shots were recorded with ~760 containing hits (1.77% hit rate) of S816–4 ApoA1 microcrystals on the bare chip. 40,500 shots were recorded with 250 containing hits for S1022 ApoE4 and 41,100 shots with 176 containing hits for M1328 ApoE4, resulting in 0.6 % and 0.4 % hit rates, respectively. Differences in hit rates are likely due to both differences in crystal density on the chip as deposited and variations in the strength of diffraction due to crystal size and quality. For each apolipoprotein and crystallization condition, diffraction was highly anisotropic but displayed distinct Bragg peaks within layer lines (Figure 4). For S816–4 ApoA1, reflections out to ~7 Å were observed for some hits although 11 Å was typical. It is likely some weak or diffuse diffraction was obscured by the low-angle attenuator. Diffraction from S1022 and M1328 ApoE4 crystals have similar characteristics in that Bragg peaks within layer lines are generally more diffuse than for ApoA1 and reflections along the direction of most diffraction did not exceed 9 Å (Figure 5).

The hits appear to have a very limited range of orientations about at least one crystallographic axis, which is consistent with a rod or needle-like morphology. In general, the crystals were much larger in one dimension than the chip pore diameter, as evident from optical microscopy of crystals deposited onto micropatterned chips prior to beamtime. Attempts at indexing the patterns failed likely due to the sparsity, low-resolution, and diffuseness of the reflections. Thus, two unit cell dimensions were approximated by measuring the reflection spacing along putative zero-level axes and parallel axes over many of the recorded hits. For approximately 30% of the available hits for S816–4 ApoA1, signal intensities along layer lines and another clear axis were integrated over a width of 3 pixels and inter-peak distances were determined. These spacings of the reciprocal lattice points were translated into real space and histograms of the resulting unit cell values were fit to determine average values, in this case 73.0 ± 7.6 Å and 38.5 ± 4.8 Å . Fewer hits with less well-defined peak positions were available for S1022 and M1328 ApoE4, thus approximately 15 individual measurements of reflection spacing were simply averaged. Analogous values obtained were 90.2 ± 10.2 Å and 41.6 ± 5.5 Å for S1022 ApoE4 and for 111.4 ± 12.1 Å and 40.8 ± 6.7 Å M1328 ApoE4. During SFX experiments at CXI in vacuum, approximately 60,000 shots were recorded for S816–4 ApoA1 microcrystals. While hit finding was somewhat hampered by the prevalence of diffraction from what appear to be partially dried buffer components, at least 100 frames show hits with identifiable NLP diffraction similar to data collected at MFX. Using similar methods and assumptions for hits analyzed from the MFX experiment for the CXI experiment, unit cell parameters of 68.4 ± 6.9Å and 36.9 ± 5.3Å were determined from 21 individual measurements of reflection spacing.

All crystallization conditions measured at MFX have strong diffuse scattering features at 4.2 Å in the direction perpendicular to the direction of strongest diffraction. These features are similar to those exhibited by previous studies of stacked lamellar membranes of DPPC at room temperature^70, 71^ and are attributed to partial ordering of the phospholipids in the form of packing distances between the hydrocarbon tails. This is a strong indication of the presence of a DMPC lipid bilayer in the NLP crystals. One of the measured unit cell dimensions that is fairly consistent across apolipoprotein and crystallization conditions is also consistent with the thickness of individual NLPs as measured by AFM^64^ as well as the thickness of a DMPC bilayer at room temperature (3.6 nm). The other unit cell dimension increases for both ApoE4 conditions compared to ApoA1, which is consistent with a larger-diameter disc as previously observed.^27^

The anisotropy of the measured diffraction and the diffuse nature of some reflections within layer lines is suggestive of fiber-like diffraction. NLPs containing neutral zwitterionic phosphocholine lipids as well as discoidal HDL and LDL are known to form “rouleaux”, where disc bilayers associate to form lamellar-like columnar structures, under certain staining and deposition conditions for negative-stain TEM, where the negatively charged stain is thought to associate with the positive surface charge of the choline head group and allow the bilayer surfaces to come together.^72^ This effect is enhanced in the presence of monovalent salt conditions that are reproduced in many of our crystallization conditions and may contribute to fibril formation. Diffraction with very similar characteristics was reported for synchrotron crystallography of ApoE4 single crystals, but with slightly more well-defined Bragg spots particularly compared to SFX results for the same apolipoprotein, possibly due in part to cryo cooling.^33, 72^

While diffraction observed in the SFX experiment is similar to results obtained from previous synchrotron cryo-crystallography experiments, SFX enables collection of effectively damage-free data^36, 37, 73^ that would not otherwise be achievable at room temperature, especially for radiation damage prone proteins as lipid-bound lipoproteins have been noted to be.^34^ Radiation damage makes room temperature data collection particularly difficult for small or needle-like crystals (with two small size dimensions), which is circumvented by both the small XFEL beam focus and short pulse duration. Moreover, room temperature data collection facilitates dynamic studies of membrane protein function, maturation processes associated with lipid and cholesterol binding to apolipoproteins, and functional processes of crystallized NLP-encapsulated membrane proteins. The capability for room temperature measurement also represents the major advantage of this approach over cryoEM for nanodisc-encapsulated membrane protein structure determination, which has recently seen great success.^2–8^ Cell-free expression and assembly of membrane proteins into NLPs and other nanodiscs is equally amenable to detergent-free structural studies with cryoEM, though this is not currently widely implemented, but in that case all dynamics must be inferred from an equilibrium conformational ensemble. Some time resolved capabilities are being developed (ref) but the time resolution is still restricted to the ~10s-100s of millisecond freezing time,^74–78^ where the SFX time resolution is only limited by the XFEL pulse duration and the duration of the trigger (substrate mixing, laser pulse duration, etc).

## 4. Summary

We present robust crystallization conditions for multiple types of lipid-bound apolipoproteins produced via cell-free expression. This study represents a broad search of the crystallization chemical space that will inform further optimization for membrane proteins supported in NLP scaffolds produced via cell-free expression. Our unit cell calculations are consistent with the presence of a discoidal structure containing a centralized DMPC lipid bilayer surrounded by apolipoproteins for these self-assembled NLPs produced with cell-free expression in a detergent-free process. Importantly, the diffraction pattens from cell-free lipid-bound apolipoproteins on fixed stages at CXI and MFX produced similar diffraction characteristics to previously published studies using more conventional NLP assembly procedures,^33, 34^ validating our approach for structural studies of detergent-sensitive membrane protein-nanodisc complexes. This is the first study to take advantage of SFX at the LCLS with low-background fixed-target sample delivery with high rep rate scanning for studying these types of nanoparticles, containing both forms of lipid bound apolipoproteins. The combined low sample consumption, low background, minimal crystal size requirements, and lack of radiation damage effects on measurement make fixed target SFX an ideal approach for characterization of the diffuse NLP diffraction, while room temperature measurement enables future dynamic studies. The crystallization protocols outlined will enable investigation of the maturation processes associated with lipid and cholesterol binding, while also potentially enabling the dynamic study of membrane bound proteins within NLPs.

## Supplementary Material

Supplementary Information

## Figures and Tables

**Table 1.** Compositional characteristics, crystal sizes, and approximate crystal densities for crystallization conditions reproduced at LLNL and chosen to optimize for SFX experiments to maximize reproducibility, crystal uniformity, and crystal density for ApoA1 and ApoE4. Boxed conditions were measured in SFX experiments at LCLS.

	Cocktail number	Buffer concentration and pH	PEG precipitant concentration	Salt additive concentration	Length range, μm (Average)	Estimated density, #crystals/μl
***ApoE4 NLPs***	M1328	0.1M Tris pH 8.5	22.6% w/v PEG 4000	0.2M Lithium Sulfate monohydrate	50–140 (85)	73

	M318	0.1M Sodium Citrate pH 5.6	16% w/v PEG 4000	0.1M Ammonium Phosphate-dibasic	50–150 (70)	17.5
	M548	0.2M Tris pH 8.5	35% w/v PEG 4000	0.2M Lithium Sulfate monohydrate	25–50 (42)	8
	M750	0.1M Tris pH 8.5	40% w/v PEG 1000	0.2M Lithium Sulfate monohydrate		
	M752	0.1M Tris pH 8.5	37.5% w/v PEG 1000	0.3M Lithium Sulfate monohydrate		

	S1022	0.1M Sodium Citrate pH 5.5	20% w/v PEG 1000	0.1M Lithium sulfate monohydrate	40–130 (52)	74

	S1438	---	22.6% w/v PEG 4000	0.2M Sodium Citrate tribasic dihydrate		
***ApoA1 NLPs***	S714	0.1M Tris pH 8.0	20% w/v PEG 1000	0.1M Magnesium chloride-hexahydrate		
	S789	0.1M CAPS, pH 10	20% w/v PEG 1000	0.1M Lithium chloride		
	S816	0.1M CAPS pH 10	20% w/v PEG 1000	0.1M Sodium bromide	80–200 (120)	84
	S816–2	0.1M CAPS pH 10	25% w/v PEG 1000	0.1M Sodium bromide	20–140 (58)	229
	S816–3	0.1M CAPS pH 10	30% w/v PEG 1000	0.1M Sodium bromide	30–100 (62)	198

	S816–4	0.1M CAPS pH 10	35% w/v PEG 1000	0.1M Sodium bromide	20–160 (75)	92

	S831	0.1M CAPS pH 10	20% w/v PEG 1000	0.1M Lithium sulfate-monohydrate		
