# Supplementary material for: Crystallization of ApoA1 and ApoE4 nanolipoprotein particles and initial XFEL-based structural studies
Source: Crystals (Basel). Author manuscript; Available in PMC 2022 Jun 8. (PMC9175823; doi:10.3390/cryst10100886)

*Crystallization of ApoA1 and ApoE4 nanolipoprotein particles for XFEL-based structural studies*

M.L. Shelby,^1^ D. Gilbile,^2^ Grant, T.D.,^3,4^ Bauer, W.J.,^4^ Segelke, B.,^1^ He W.,^1^ Evans A.C.,^1,2^ Crespo, N., ^3,4^ Fischer, P.,^5^ Pakendorf, T.,^5^ Hennicke, V.,^5^ Hunter, M.S.,^6^ Batyuk, A.,^6^ Barthelmess M.,^5^ Meents, A.,^5^ Kuhl, T.,^2^ Frank M.,^1,2^ and Coleman, M.A.,^1,2^

^1^Biosciences and Biotechnology Division, Lawrence Livermore National Laboratory, Livermore, CA, USA.

^2^Department of Chemical Engineering, University of California at Davis, Davis, CA, USA.

^3^Department of Structural Biology, Jacobs School of Medicine and Biomedical Sciences, SUNY University at Buffalo, Buffalo, NY, USA

^4^Hauptman-Woodward Medical Research Institute, Buffalo, NY, USA.

^5^Center for Free-Electron Laser Science, Hamburg, Germany.

^6^Linac Coherent Light Source, SLAC National Accelerator Laboratory, Menlo Park, California, USA.

***Figure S1:*** General design of two styles of chips used in experiments at MFX in near 100% relative humidity (A) and the Graphene/PMMA enclosed chips used at CXI in vacuum (B). Chips in (A) utilized a 3 x 1 large-window layout with 100 µm hexagonal pore spacing and samples were measured with no enclosing layers. Chips in (B) utilized an 18 x 5 small-window layout with 50 µm hexagonal pore spacing and were enclosed with Graphene/PMMA films supported by the chip and a Kapton frame.


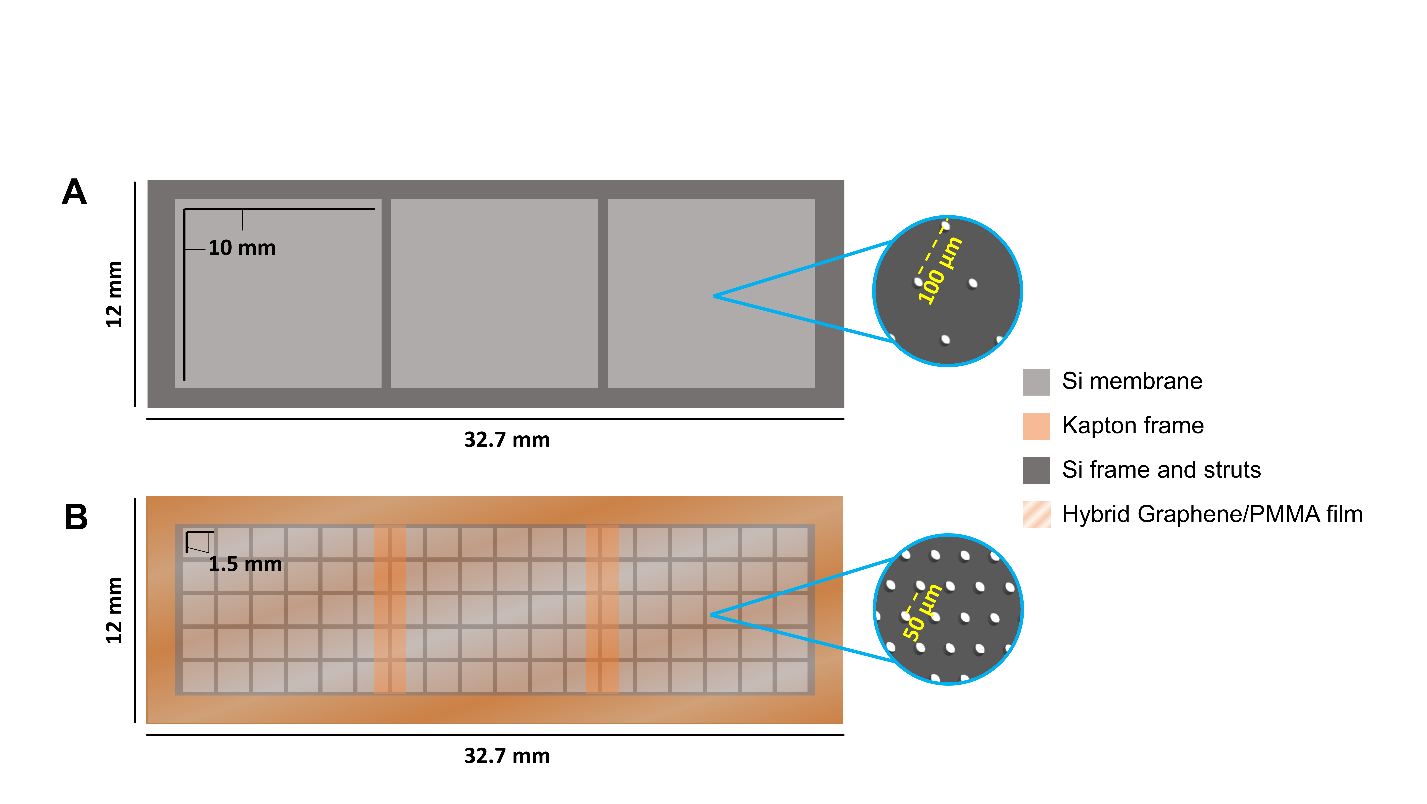


***Figure S2:*** Optical microscopy image showing NLP crystals deposited on a single Graphene/PMMA film supported by the hexagonal pore patterned Si membrane of the chip and enclosed in a second Graphene/PMMA film. After enclosure, the assembly was exposed to vacuum for 30 min without any apparent desiccation of the enclosed sample. Rod-shaped crystals are circled in white.
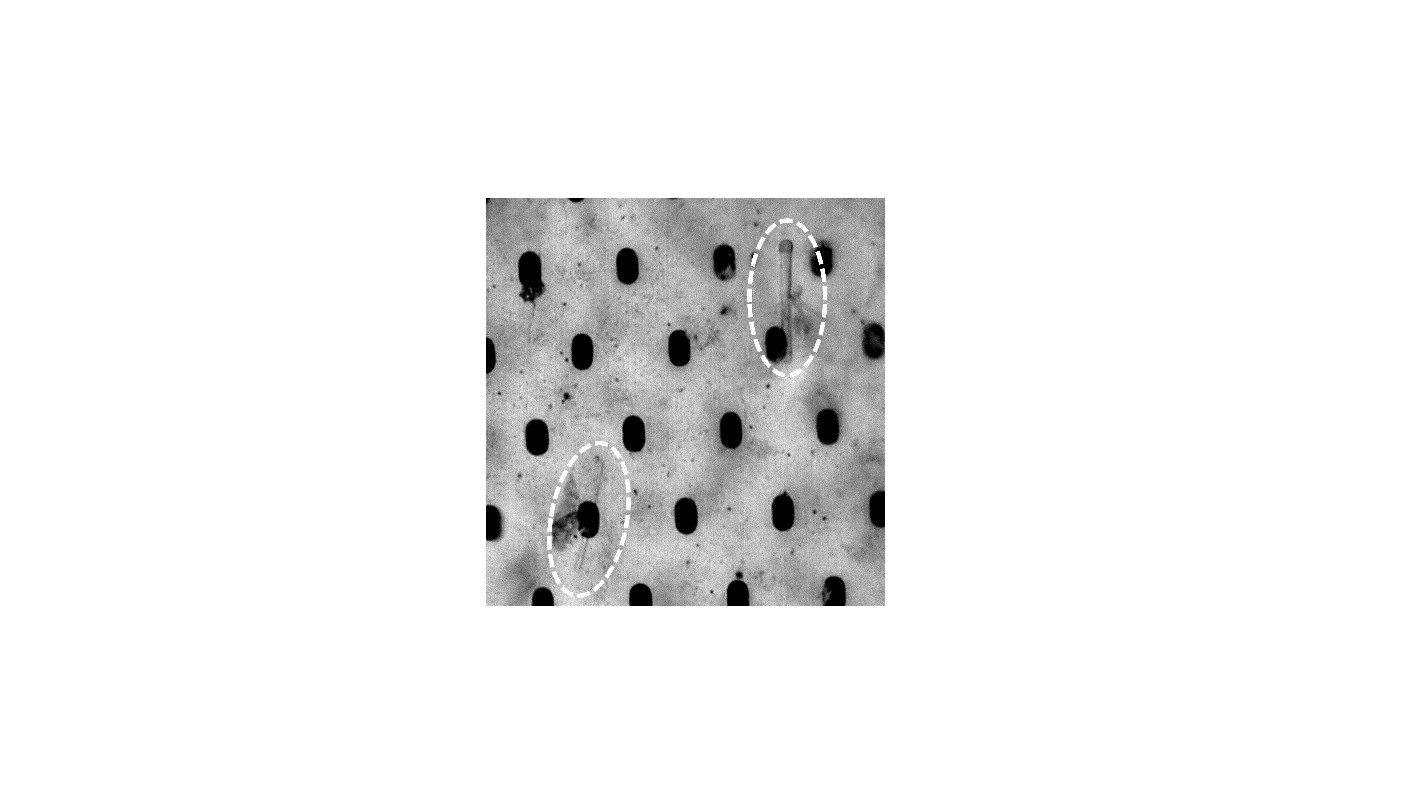


***Figure S3:*** Size distributions of ApoA1 (A) and ApoE4 (B) NLP hydrodynamic radii as measured by dynamic light scattering (DLS)
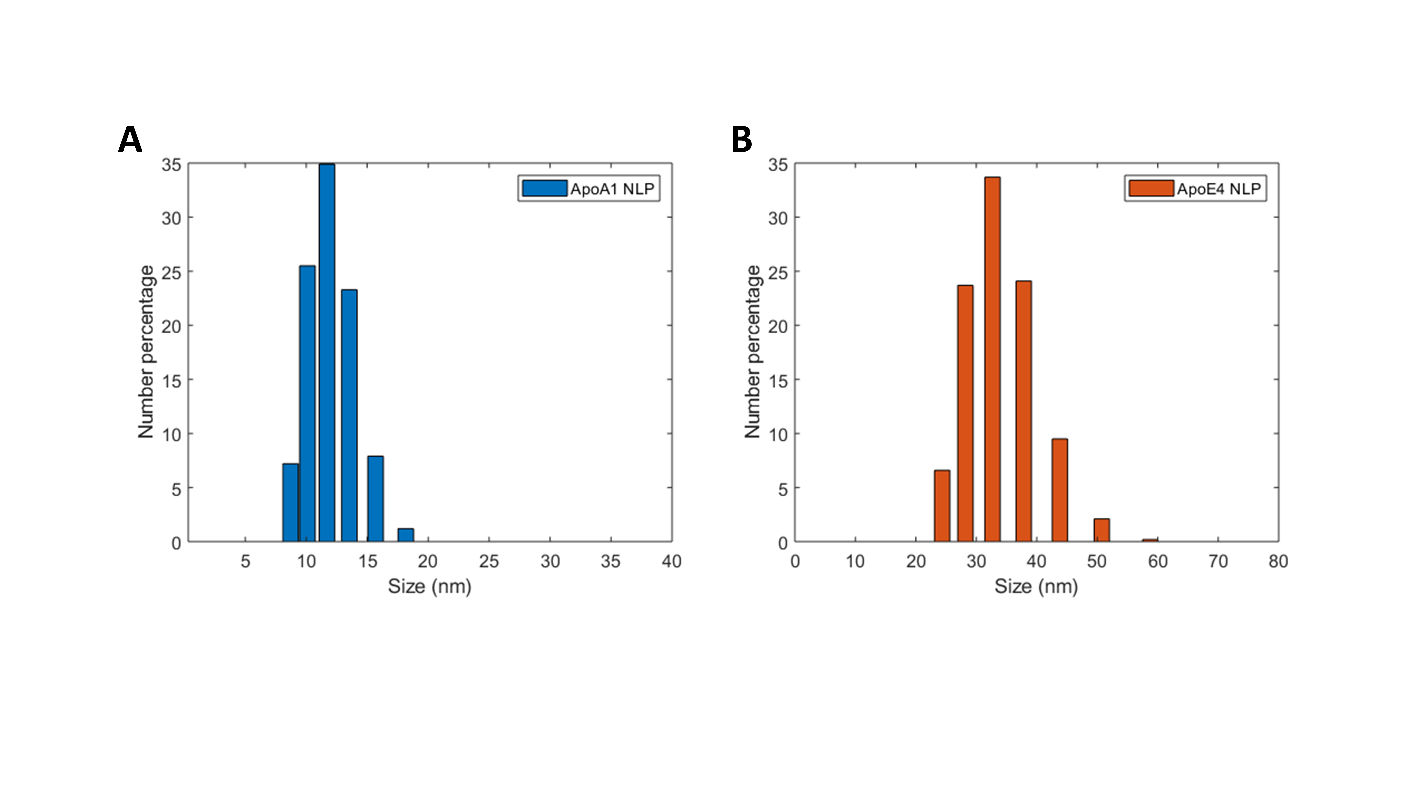


***Table S1*.** Salt composition for all hits from the HWI soluble and membrane screens for ApoA1 and ApoE4.

|  |  | ***ApoA1 NLPs*** | | | | ***ApoE4 NLPs*** | | | |
| --- | --- | --- | --- | --- | --- | --- | --- | --- | --- |
| **CATION** | **charge** | **Total hits (out of 165)** | **% of total hits** | **total cocktails containing this component** | **% of hits in total cocktails containing this component** | **Total hits (out of 237)** | **% of hits** | **total cocktails containing this component** | **% of hits in total cocktails containing this component** |
| Magnesium | +2 | 57 | 34.55 | 330 | 17.27 | 123 | 51.90 | 394 | 31.21 |
| Sodium | +1 | 49 | 29.70 | 1186 | 4.13 | 58 | 24.47 | 1191 | 4.86 |
| Ammonium | +1 | 29 | 17.58 | 750 | 3.87 | 43 | 18.14 | 750 | 5.73 |
| Lithium | +1 | 13 | 7.88 | 243 | 5.35 | 21 | 8.86 | 243 | 8.64 |
| Potassium | +1 | 10 | 6.06 | 365 | 2.74 | 13 | 5.49 | 365 | 3.56 |
| Calcium | +2 | 6 | 3.64 | 117 | 5.13 | N/A | N/A | N/A | N/A |
| Barium | +2 | 1 | 0.61 | 1 | 100.00 | N/A | N/A | N/A | N/A |
| Rubidium | +1 | 1 | 0.61 | 25 | 4.00 | N/A | N/A | N/A | N/A |
| ANION | charge | Total hits (out of 165) | % of total hits | total cocktails | % hits/total cocktails | Total hits (out of 237) | % of hits | total cocktails | % hits/total cocktails |
| Chloride | -1 | 89 | 53.94 | 1011 | 8.80 | 136 | 57.38 | 1074 | 12.66 |
| Sulfate | -2 | 26 | 15.76 | 570 | 4.56 | 42 | 17.72 | 570 | 7.36 |
| Citrate | -3 | 24 | 14.55 | 541 | 4.44 | 41 | 17.30 | 541 | 7.57 |
| Phosphate | -3 | 15 | 9.09 | 408 | 3.68 | 22 | 9.28 | 408 | 5.39 |
| Bromide | -1 | 5 | 3.03 | 101 | 4.95 | 1 | 0.42 | 101 | 0.99 |
| Acetate | -1 | 4 | 2.42 | 316 | 1.27 | 5 | 2.11 | 318 | 1.57 |
| Nitrate | -1 | 4 | 2.42 | 111 | 3.60 | 3 | 1.27 | 111 | 2.70 |
| Carbonate | -2 | 3 | 1.82 | 26 | 11.54 | N/A | N/A | N/A | N/A |
| Molybdate | -2 | 2 | 1.21 | 20 | 10.00 | 2 | 0.84 | 20 | 10 |
| Thiocyanate | -1 | N/A | N/A | N/A | N/A | 1 | 0.42 | 50 | 2 |
| Thiosulfate | -2 | N/A | N/A | N/A | N/A | 1 | 0.42 | 14 | 7.14 |

***Figure S4*.** Crystallization buffer conditions and polymer precipitant composition for all hits from the HWI soluble and membrane screens for ApoA1 and ApoE4.


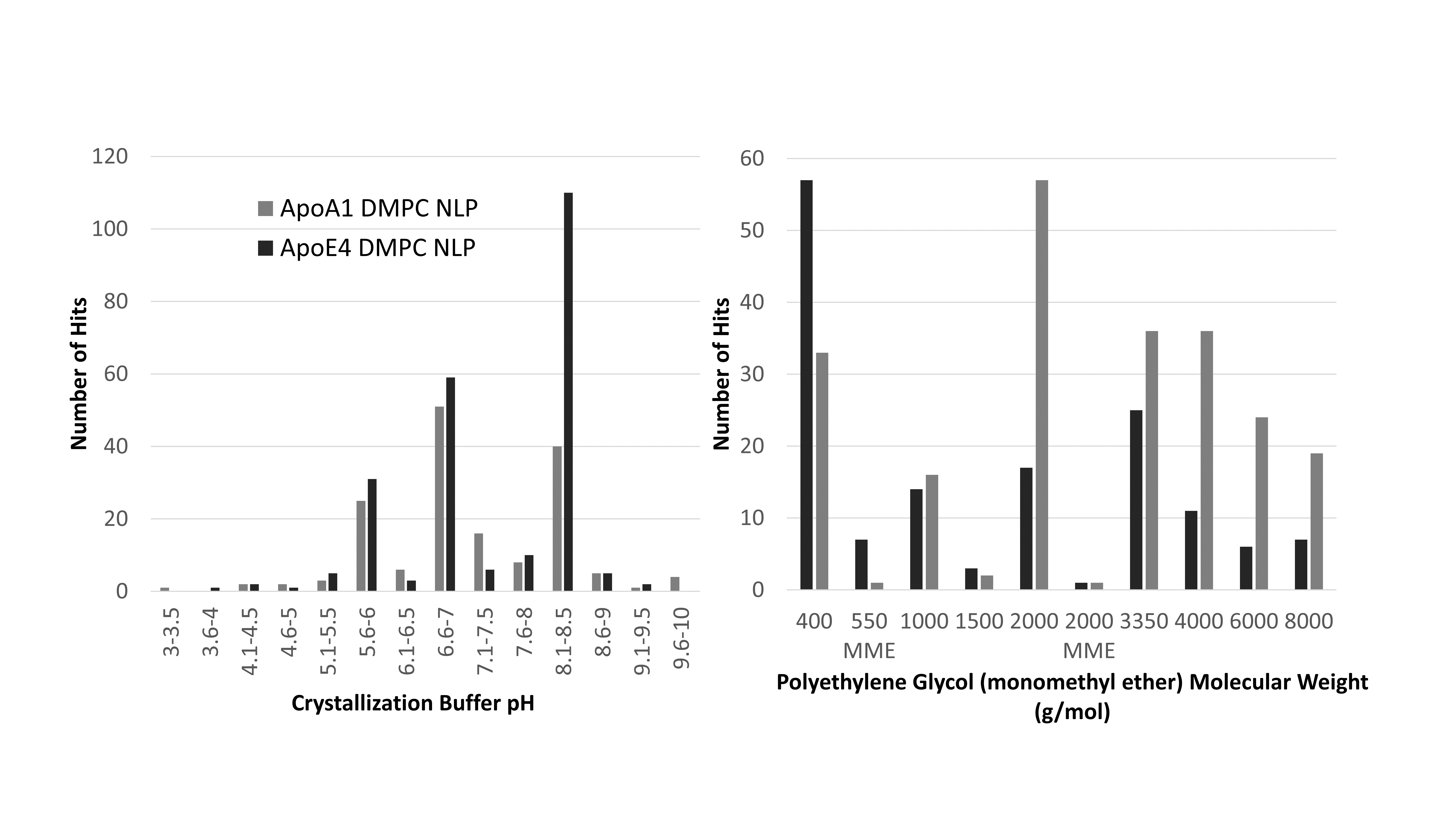

Supplement: Supplementary Information [file NIHMS1751963-supplement-Supplementary_Information.docx]
